# Exploring the Influence of *VDR* Genetic Variants *TaqI*, *ApaI*, and *FokI* on COVID-19 Severity and Long-COVID-19 Symptoms

**DOI:** 10.3390/jpm13121663

**Published:** 2023-11-28

**Authors:** Ghayda’ Alhammadin, Yazun Jarrar, Abdalla Madani, Su-Jun Lee

**Affiliations:** 1 Department of Pharmaceutical Science, College of Pharmacy, Al-Zaytoonah University of Jordan, Amman 11733, Jordan; 202027028@std-zuj.edu.jo (G.A.); abdulla.elmadani@zuj.edu.jo (A.M.); 2 Department of Basic Medical Sciences, Faculty of Medicine, Al-Balqa Applied University, Al-Salt 19117, Jordan; yazan.jarrar@bau.edu.jo; 3 Department of Pharmacology and Pharmacogenomics Research Center, College of Medicine, Inje University, Busan 50834, Republic of Korea

**Keywords:** COVID-19, Jordanians, long-COVID-19 symptoms, *VDR* variants

## Abstract

There is increasing evidence regarding the importance of vitamin D in the prognosis of coronavirus disease 2019 (COVID-19). Genetic variants in the vitamin D receptor (*VDR*) gene affect the response to vitamin D and have been linked to various diseases. This study investigated the associations of the major *VDR* genetic variants *ApaI*, *FokI*, and *TaqI* with the severity and long post-infection symptoms of COVID-19. In total, 100 Jordanian patients with confirmed COVID-19 were genotyped for the *VDR ApaI*, *FokI*, and *TaqI* variants using the polymerase chain reaction-restriction fragment length polymorphism (PCR-RFLP) method. COVID-19 severity, the most commonly reported long-COVID-19 symptoms that lasted for >4 weeks from the onset of infection, and other variables were analyzed according to *VDR* genetic variants. In this study, *ApaI* and *FokI* polymorphisms showed no significant associations with COVID-19 severity (*p* > 0.05). However, a significant association was detected between the *TaqI* polymorphism and the severity of symptoms after infection with the SARS-CoV-2 virus (*p* = 0.04). The wild-type *TaqI* genotype was typically present in patients with mild illness, whereas the heterozygous *TaqI* genotype was present in asymptomatic patients. With regard to long-COVID-19 symptoms, the *VDR* heterozygous *ApaI* and wild-type *TaqI* genotypes were significantly associated with persistent fatigue and muscle pain after COVID-19 (*p* ˂ 0.05). Most carriers of the heterozygous *ApaI* genotype and carriers of the wild-type *TaqI* genotype reported experiencing fatigue and muscle pain that lasted for more than 1 month after the onset of COVID-19. Furthermore, the *TaqI* genotype was associated with persistent shortness of breath after COVID-19 (*p* = 0.003). Shortness of breath was more common among individuals with homozygous *TaqI* genotype than among individuals with the wild-type or heterozygous *TaqI* genotype. *VDR TaqI* is a possible genetic variant related to both COVID-19 severity and long-COVID-19 symptoms among Jordanian individuals. The associations between *VDR TaqI* polymorphisms and long-COVID-19 symptoms should be investigated in larger and more diverse ethnic populations.

## 1. Introduction

The coronavirus disease 2019 (COVID-19) pandemic has been associated with substantial morbidity and mortality, along with profound effects on daily life worldwide. COVID-19 is caused by severe acute respiratory syndrome coronavirus 2 (SARS-CoV-2), which has the potential to disrupt multiple systems, including the respiratory, gastrointestinal, musculoskeletal, and neurological systems [1]. The clinical symptoms of COVID-19 vary from asymptomatic to severe, ranging from pneumonia to acute respiratory disease syndrome and multiorgan dysfunction [2]. Some patients may experience symptoms lasting over 4 weeks following the onset of COVID-19, which is also known as persistent or long-lasting COVID-19. A previous meta-analysis showed that more than 60% of patients with COVID-19 have one or more persistent symptoms lasting over 1 month after disease onset [3]. The most frequently reported long-COVID-19 symptoms are fatigue, muscle pain, joint pain, cough, shortness of breath, palpitations, and chest pain [4].

Vitamin D (VD) has immunomodulatory, antioxidant, and anti-inflammatory effects [5]. Moreover, VD can increase resistance to infection through numerous mechanisms, including stimulating the release of defensins, which help combat pneumonia by reducing viral replication rates, enhancing the synthesis of anti-inflammatory cytokines, and decreasing concentrations of proinflammatory cytokines [6]. These mechanistic findings have been supported by several clinical studies, which showed that VD deficiency increases the likelihood of acute viral respiratory tract infection in both adults and children [7].

VD exerts its effect after binding to the nuclear vitamin D receptor (VDR) and stimulating the VD/VDR signaling pathway [8]. Therefore, VDR dysregulation may result in various diseases and autoimmune conditions [9,10]. The *VDR* gene, which encodes the VDR, lies on human chromosome 12q13.11 [11]. Genetic variants, such as single-nucleotide polymorphisms, that affect the gene encoding the VDR can reduce VD activity and have been linked to a variety of diseases, including rheumatoid arthritis, asthma, and susceptibility to tuberculosis and infections with enveloped viruses [12,13,14]. *ApaI*, *FokI*, and *TaqI* are the best-studied *VDR* genetic variants [11]. *FokI* and *TaqI* have impacts on the translation and structure of the VDR; *ApaI* is linked to changes in its mRNA stability and decreased levels of expression. All of these genetic variants lead to altered VDR activity, thus impacting the main effects of VD in the body [15].

One previous review investigated the importance of VD in the prognosis of COVID-19 symptoms and showed that VD deficiency was correlated with COVID-19-induced acute respiratory disease syndrome [16]. However, there have been few genetic investigations regarding the prevalence of *VDR* genetic variants and their effects on COVID-19 prognosis. Among patients with both mild/moderate and severe/critical COVID-19, *VDR* genetic variants exhibited substantial relationships with various clinical outcomes, including disease severity and shortness of breath. In one study, eight *VDR* polymorphisms were genotyped for 500 hospitalized patients with COVID-19 in Iran; six *VDR* polymorphisms displayed significant associations with disease severity and shortness of breath in both mild/moderate and severe/critical disease groups [17]. Another study investigated links between COVID-19 prognosis and *VDR FokI*, *ApaI*, *TaqI*, and *BsmI* genotypes in Turkey. The findings revealed independent associations of *VDR* polymorphisms with disease severity and mortality after SARS-CoV-2 infection; moreover, the wild-type *Taq* genotype was common among patients admitted to the intensive care unit [18].

To our knowledge, there have been no reports regarding the associations of *VDR* genetic polymorphisms with long-COVID-19 symptoms. However, the results of some studies suggested links with fatigue and muscle pain, as well as impaired lung function that can ultimately cause shortness of breath [19,20]. The present study assessed the associations of *VDR ApaI*, *FokI*, and *TaqI* genotypes with the severity of symptoms experienced during SARS-CoV-2 infection and long-COVID-19 symptoms lasting over 4 weeks from the onset of infection in the Jordanian population. The findings provide novel insights into the associations of distinct genetic variants with the etiology and pathogenesis of COVID-19. Moreover, this study might contribute to comprehending the impact of genetic polymorphisms in the vitamin D receptor with long-term symptoms that could be induced by other viruses similar to COVID-19, and help be more prepared to provide the appropriate medical care and treatment for those patients.

## 2. Materials and Methods

### 2.1. Chemicals

Polymerase chain reaction (PCR) primers were designed and acquired from Integrated DNA Technologies (Coralville, IA, USA). The primer sequences, based on a previously published protocol [21], are presented in Table 1. The Wizard Genomic DNA Purification Kit (cat. no. A1120), PCR master mix, and 100-base pair (bp) ladder were obtained from Promega Corp. (Madison, WI, USA). 10× Tris–ethylenediaminetetraacetic acid–borate buffer and agarose gel electrophoresis system were acquired from Bio Basic (Markham, ON, Canada). RedSafe dye was obtained from iNtRON Biotechnology (Seongnam, Republic of Korea). For the PCR-RFLP analysis, the restriction enzymes *FokI* (cat. no. R0109S) and *TaqI*-v2 (cat. no. R0149S) were obtained from New England Biolabs (Ipswich, MA, USA); *ApaI* (cat. no. R6361) was purchased from Promega Corp.

### 2.2. Participants

In total, 100 patients with COVID-19 (68 women and 32 men, aged 37.39 ± 13.8 years) were enrolled in the study. Blood samples were collected from randomly selected participants at High-Quality Laboratories (Madaba, Jordan) with the assistance of licensed technicians. The participants had previously been infected with COVID-19 between February 2022 and December 2021. Subsequently, the threat of the COVID-19 pandemic significantly diminished in Jordan. Data collection and blood samples for *VDR* genotyping took place from 1 December 2022 to 25 February 2023. Consequently, persistent symptoms may have manifested before the commencement of this study in December 2022. None of the participants in this study had been infected with COVID-19 in close proximity to the study’s initiation. COVID-19 statuses were reported to the Jordanian Ministry of Health in 2022 and 2023 based on a positive result in real-time (RT)-PCR analysis of pharyngeal or nasal swabs. The exclusion criteria were as follows: non-Jordanian ethnicity, age < 18 years, and lack of COVID-19 status registration at the Jordanian Ministry of Health. All participants were unrelated to each other. The ethnicities of the participants were determined as self-reported by each participant. The researchers then checked the paternal and maternal family names of each participant which can also help in excluding participants of non-Jordanian ethnicity. All participants provided written informed consent to take part in the study. The study protocol was approved by the University of Al-Zaytoonah (approval no. 2022-2021/13-3).

### 2.3. Data Collection

A self-report questionnaire was designed, consisting of eight questions regarding three topics. The first section recorded demographics information, including age, sex, body mass index (BMI), and smoking status. Weight categories were determined based on BMI [22]. Smoking statuses were determined using the pack-year indicator which quantifies the total number of cigarettes smoked by an individual throughout their lifetime (one pack is defined as 20 cigarettes) [23]. The second section explored the presence of any comorbidities, and the third section examined the severity of prior COVID-19 based on the last update of the COVID-19 Treatment Guideline in August 2022 [24]. This guideline divides COVID-19 severity into five stages: asymptomatic, mild, moderate, severe, and critical. It also defines long-COVID-19 as symptoms lasting greater than 4 weeks. The authors translated all questions to Arabic and presented them to participants in an easy-to-understand manner; responses were selected using a multiple-choice method.

### 2.4. Genotyping of VDR Gene Polymorphisms by PCR-RFLP Method

Samples of ~5 mL of peripheral blood were collected from each participant into tubes containing ethylenediaminetetraacetic acid. DNA was extracted using a Wizard Genomic DNA Purification Kit (Promega Corp.), in accordance with the manufacturer’s instructions. Before amplification of the 100 DNA samples using a T100 thermal cycler (Bio-Rad, Hercules, CA, USA), the PCR protocol was modified to establish the ideal DNA template concentration and volumes of PCR components, including forward and reverse primers, master mix, and nuclease-free water; we also determined the appropriate annealing and elongation temperatures for *VDR* gene sequence amplification, while avoiding nonspecific products, excessive smearing, and primer dimer formation. We performed two rounds of PCR. In the first round of PCR, 100 ng of genomic DNA were added to a reaction mixture consisting of 5 pmol each of *FokI* forward and reverse primers, 15 µL of green 2× One Taq Master Mix, and nuclease-free water to a final volume of 30 µL. The reaction was performed with an initial denaturation step at 94 °C for 4 min, followed by 35 cycles of denaturation at 94 °C for 45 s, annealing at 57 °C for 1 min, and elongation for 1 min at 72 °C; the final extension step comprised incubation at 72 °C for 10 min. In the second round of PCR, the total volume of 50 µL contained 100 ng of genomic DNA, 5 pmol of *ApaI-TaqI* forward primers, 5 pmol of *ApaI-TaqI* reverse primers, 25 µL of green 2× One Taq Master Mix, and 22.5 µL of nuclease-free water. The reaction was performed with an initial denaturation step at 94 °C for 4 min, followed by 40 cycles of denaturation at 94 °C for 45 s, annealing at 55 °C for 1 min, and elongation for 1 min at 72 °C; the final extension step comprised incubation at 72 °C for 10 min. For confirmation of the amplification process, the PCR amplicons were separated by electrophoresis in a 2% (*w/v*) agarose gel at 125 V for 30 min. Bands on the gel were visualized using a tabletop ultraviolet transilluminator (BioDoc-Itt; Antylia Scientific, Vernon Hills, IL, USA). The predicted sizes of the *VDR ApaI-TaqI* and *FokI* fragments were 745 bp and 245 bp, respectively.

The target *VDR* DNA fragments were digested using appropriate restriction enzymes under optimized conditions. The first enzymatic digestion was performed in a mixture containing 10 µL of *ApaI-TaqI* PCR product, 2 µL of RE 10× Buffer A (cat. no. R001A, Promega Corp, Madison, WI, USA), 10 units of *ApaI*, 0.1 µL of acetylated bovine serum albumin (R396D, Promega Corp, Madison, WI, USA), and 7 µL of nuclease-free water; the mixture was incubated at 37 °C for 90 min, then subjected to heat inactivation at 65 °C for 20 min. The second enzymatic digestion was performed in a mixture containing 10 µL of *FokI* PCR product, 2 µL of 10× NEBuffer (New England Biolabs, Ipswich, MA, USA), 10 units of *FokI* (R0109S, New England Biolabs, Ipswich, MA, USA), and 7 µL of nuclease-free water; the mixture was incubated at 37 °C for 4 h, then subjected to heat inactivation at 65 °C for 20 min. The third enzymatic digestion was performed in a mixture containing 10 µL of *ApaI-TaqI* PCR product, 2 µL of 10× NEBuffer, 10 units of *TaqI-v2* (R6361, New England Biolabs, Ipswich, MA, USA), and 7 µL of nuclease-free water; the mixture was incubated at 65 °C for 45 min, then subjected to heat inactivation at 80 °C for 20 min. The digestion products were separated by 3.5% agarose gel electrophoresis at 125 V for 55 min. The genotypes of all polymorphisms were identified based on the digest patterns (Supplementary Figure S1a–c).

### 2.5. Statistical Analysis

Statistical analyses were performed using SPSS version X7 (IBM SPSS Inc., Armonk, NY, USA). The frequencies of categorical variables were compared using the chi-square test. A multiple logistic regression analysis using the chi-square was conducted, incorporating independent variables, in order to identify factors associated with severity of COVID-19 symptoms. In all analyses, *p* < 0.05 was assumed to indicate statistical significance. The allele frequencies of each of the *VDR* genetic variants were determined using Chi-square (*χ*^2^) test and the odds ratio and 95% confidence intervals (CIs) were reported for the significant comparison. Deviation from the Hardy–Weinberg equation was tested using a Chi-square test comparing the observed and expected frequency of VDR genotypes.

## 3. Results

### 3.1. Baseline Characteristics of Patients

Table 2 shows the demographic data for all individuals with COVID-19, including sex, age, BMI, and smoking status. More than half of the patients (55%) exhibited normal weight; the remaining patients were classified as overweight (36%), underweight (6%), or obese (3%). Most patients (70%) were nonsmokers; 19%, 9%, and 2% were light, moderate, and heavy smokers, respectively. With regard to comorbidities among the patients, 13% had type 2 diabetes, 11% had hypertension, and 5% had chronic vascular disease. Additionally, 4.4% of the female patients were pregnant while they had COVID-19. None of the patients had renal, liver, or lung diseases (Table 2).

### 3.2. Severity of COVID-19 Symptoms, SARS-CoV-2 and Long-Lasting Symptoms among COVID-19 Infected Individuals

As shown in Table 3, half of the patients in this study did not show any COVID-19 symptoms while infected with SARS-CoV-2. The majority of the asymptomatic individuals had heterozygous *ApaI*, wild-type *FokI*, and wild-type *TaqI* genotypes. Among the remaining patients (50% total), 48% experienced mild illness, 1% experienced moderate illness, and 1% experienced severe illness. None of the participants had critical illness with respiratory failure. The prevalences of long-COVID-19 symptoms (present for >4 weeks after acute COVID-19) were 27% for fatigue and muscle pain, 22% for joint pain, 8% for cough, 7% for shortness of breath, 3% for chest pain, and 2% for cardiac palpitations.

### 3.3. VDR Genotypes of Patients with COVID-19

The frequencies of the *VDR ApaI*, *FokI*, and *TaqI* genotypes among patients with COVID-19 are shown in Table 4. The prevalences of wild-type, heterozygous, and homozygous *ApaI* genotypes were 36.0%, 45.0%, and 19.0%, respectively. The observed allele frequency of *ApaI* A > C was 41%. *FokI* genotypes were identified as wild-type, heterozygous, and homozygous in 62.0%, 28.0%, and 10.0% of patients, respectively. The observed allele frequency of *FokI* C > T was 24%. The frequencies of wild-type, heterozygous, and homozygous *TaqI* genotypes were 54.0%, 32.0%, and 14.0%, respectively. The observed allele frequency of *TaqI* T > C was 24%. The allele frequencies of all of the tested *VDR* genetic variants were in Hardy–Weinberg equilibrium, with *p*-values > 0.05 (Chi-square test).

### 3.4. VDR Haplotype and Linkage Disequilibrium among COVID-19 Infected Individuals

**Figure 1:**
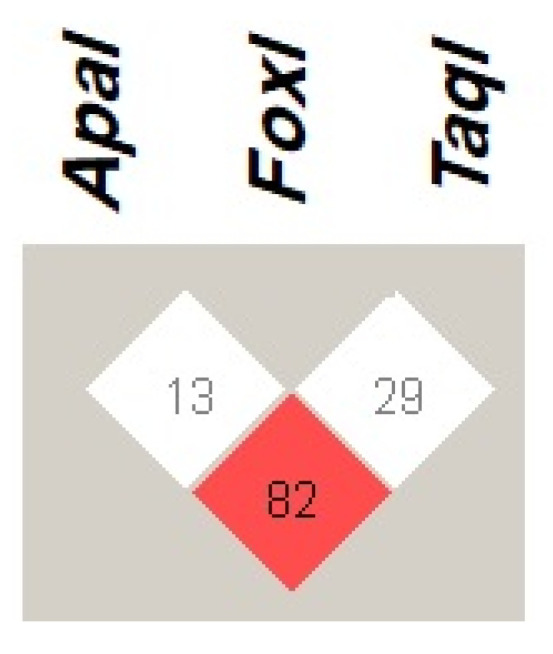
Linkage disequilibrium (LD) of VDR genetic variants *ApaI* (rs7975232 A > C), *FokI* (rs2228570 C > T), and *TaqI* (rs731236 T > C), among patients with COVID-19. The red square indicates a strong LD, whereas the white square indicates a weak LD between the VDR genetic variants. The number within the squares represents D’ value. Haploview 4.2 software was used to determine LD.

The *VDR* haplotype *ApaI (C)*, *FokI (C)*, and *TaqI (T)* with frequency of 28.2% was the most common observed haplotype among patients with COVID-19. The least prevalent *VDR* haplotypes was *ApaI (C)*, *FokI (T)*, and *TaqI (C)*, with frequency of less than 1% as presented in Table 5. Regarding the LD of *VDR* variants (Figure 1), the present study found that *ApaI* was in strong linkage disequilibrium (LD) with the *TaqI* variation (D’ = 82). However, the *FokI* variant did not show LD with the other investigated *VDR* variants. 

### 3.5. VDR Variants and Their Associations with COVID-19 Severity

Table 6 shows the associations of *VDR ApaI*, *FokI*, and *TaqI* genotypes with COVID-19 severity in previously infected patients. For the *ApaI* variant, the heterozygous genotype was more prevalent among asymptomatic and mildly ill patients than among patients with moderate and severe disease (22 and 22 vs. 0 and 1, respectively). For the *FokI* variant, the wild-type genotype was most common; it was detected in 31 patients with asymptomatic infection, 29 with mild illness, one with moderate illness, and one with severe illness. For the *TaqI* variant, the wild-type genotype was most common; it was present in 24, 29, and 1 patients with asymptomatic, mild, and moderate disease, respectively.

There were no significant (*p* > 0.05) associations of *ApaI* or *FokI* polymorphisms with COVID-19 severity. In contrast, the *TaqI* variant showed a possible association with COVID-19 severity (*p* = 0.045). One patient with the homozygous *TaqI* genotype experienced severe symptoms of COVID-19.

Lastly, we found in this study that patient’s age, BMI, and the comorbidity of cardiovascular diseases are other non-genetic factors associated with the severity of COVID-19 symptoms (Supplementary Table S1).

### 3.6. VDR Variants and Their Associations with Long-COVID-19 Symptoms

The present study evaluated the associations of *ApaI*, *FokI*, and *TaqI VDR* variants with various COVID-19 symptoms lasting over 4 weeks (Table 7). The *FokI* genotype was not linked to any of the listed persistent symptoms. Nevertheless, the *ApaI* and *TaqI* genotypes showed significant associations with fatigue and muscle pain lasting over 4 weeks after the initial infection. Patients with the heterozygous *ApaI* genotype had a significantly (*p* = 0.000) higher risk of fatigue and muscle pain, compared with patients exhibiting the wild-type or homozygous genotype. Patients with COVID-19 exhibiting the wild-type *TaqI* genotype showed a significantly higher risk of fatigue and muscle pain, compared with patients who had the heterozygous or homozygous genotype (*p* = 0.036). Furthermore, *TaqI* was associated (*p* = 0.003) with persistent shortness of breath after COVID-19. Carriers of the *TaqI* homozygous genotype were more likely to have shortness of breath, compared with patients exhibiting the wild-type or heterozygous genotype.

## 4. Discussion

The global COVID-19 pandemic, caused by SARS-CoV-2 infection, has been among the most devastating health emergencies in recent times. According to the Jordanian Ministry of Health, there were 1,746,997 confirmed cases of COVID-19 with 14,122 deaths reported to the World Health Organization between 3 January 2020 and 21 June 2023 [25].

There is considerable interindividual variation in COVID-19 severity, ranging from absence of symptoms to serious respiratory failure and death [26]. This variation may be related to each patient’s health status and/or genetic background [27]. Several studies have identified numerous genetic variants correlated with COVID-19 severity, including *ACE2*, *ABO*, *CD26*, *IFITM3*, *HLA*, *TLR7*, and *TMPRSS2* [28,29,30]. The results of numerous studies have suggested that VD deficiency is involved in the pathology of severe COVID-19 [16], [31]. The activities of VD are primarily regulated by its intranuclear receptor, VDR [32].

VDR expression and regulation are controlled by various processes, including VD autoregulation, transcription factors, methylation of the promoter region, and genetic variants [33]. The present study evaluated possible associations between *VDR ApaI*, *FokI*, and *TaqI* variants and COVID-19 severity based on the symptoms experienced during the period of infection, as well as long-COVID-19 symptoms lasting over 4 weeks from the onset of infection, among Jordanian patients.

Half of the participants in this study did not show any symptoms during the period of SARS-CoV-2 infection. Among patients in the other half (50% total), 48% experienced mild symptoms, 1% experienced moderate symptoms, and 1% experienced severe symptoms. None of the individuals reported a serious condition or exhibited respiratory failure during the period of infection. Consistent with our findings, some previous studies showed that many individuals were asymptomatic or had relatively mild symptoms during the period of SARS-CoV-2 infection, but they were capable of viral spread [34]. In a previous investigation, Al Harbi et al. found that 7.68% of patients with COVID-19 required urgent medical care, whereas the remaining patients (92.32%) exhibited mild to moderate illness [35]. Tabacof et al. reported a wide variety of persistent symptoms in a group of 84 individuals with previously confirmed COVID-19, which continued for an average of 151 days; the most common persistent symptoms were fatigue and muscle pain in 92% of patients, and the majority of individuals reported greater degrees of disability related to shortness of breath, tiredness, and decreased quality of life [36]. In 2020, a cross-sectional study of 430 patients in Egypt revealed that 86% experienced persistent symptoms; the most commonly reported symptoms were fatigue (60.0%), joint pain (57.2%), difficulty sleeping (50.9%), chest pain (32.6%), shortness of breath (29.1%), and cough (29.3%) [37]. Similar to our results, a previous study showed that more than 50% of participants experienced persistent fatigue during 10 weeks of monitoring after SARS-CoV-2 infection; there were no links among fatigue, COVID-19 severity, and the concentrations of inflammatory markers [38]. The occurrence of long-lasting symptoms after SARS-CoV-2 infection is frequently regarded as unexpected or unusual, but evidence suggests that it is common. Post-viral fatigue is a typical symptom of infections with the viruses causing Ebola, influenza, Middle East respiratory syndrome, and SARS [39,40].

In the present study, the *VDR* gene was genotyped by PCR-RFLP. The observations of the present study in terms of *VDR* genotype frequency, *VDR* variant allele frequency, haplotype, and LD were similar to previous findings conducted in Jordan. Khdair et al. investigated the prevalence of *VDR* genotypes and haplotypes in 100 T1DM patients and compared them to healthy volunteers [41]. In addition, Alhawari et al. analyzed the frequency of *VDR* genotypes and haplotypes in 90 T2DM patients in a Jordanian population [21]. The most prevalent *VDR* genotypes were *VDR* heterozygous *ApaI*, wild-type *FokI*, and wild-type *TaqI* (45%, 62%, and 54%, respectively). The *VDR* haplotype *ApaI (C)*, *FokI (C)*, and *TaqI (T)* was the major *VDR* haplotype in COVID-19 infected patients, as well as T2DM, T1DM patients, and healthy volunteers in Jordan. Regarding the LD of *VDR* variants, our results showed that *ApaI* is in a strong LD (D’ = 82) with *TaqI*, while *FokI* was in weak LD with other *VDR* variants, which is in line with the previous two studies.

The present study did not reveal any significant associations between *ApaI* or *FokI* genetic variants and COVID-19 severity, although *TaqI* polymorphism was associated with the occurrence of severe symptoms during the period of infection (*p* = 0.045). One patient with the homozygous *TaqI* genotype exhibited severe symptoms. The wild-type genotype was typically present in mildly ill patients, whereas the heterozygous genotype was identified in asymptomatic patients. These results were consistent with findings by Peralta et al. in a study of associations between *TaqI* polymorphisms and the likelihood of developing COVID-19 in 104 Cuban patients, which indicated that the homozygous genotype was associated with a greater risk of severe symptoms and the heterozygous genotype was mostly present in asymptomatic patients [42]. Similar to the present results, a study in Serbia showed that the *VDR FokI* variant was not associated with COVID-19 severity [43]. In contrast, Apaydin et al. reported that the *FokI* genotype was associated with severe symptoms, and there was no significant association between *TaqI* genotype and infection severity [18].

In the present study of relationships between *VDR* genetic variants and long-COVID-19 symptoms, the *FokI* genotype was not associated with persistent symptoms among individuals with a history of COVID-19 (Table 6). However, the *ApaI* and *TaqI* genotypes were significantly associated with fatigue and muscle pain lasting over 4 weeks after infection (*p* = 0.00–0.036). Additionally, *TaqI* was associated with persistent shortness of breath after SARS-CoV-2 infection (*p* = 0.003). Shortness of breath was more common among individuals with the homozygous genotype than among individuals with the wild-type or heterozygous genotype in this study.

To our knowledge, no previous reports have described the associations of *VDR* genetic variants with persistent symptoms after COVID-19. Fatigue and muscular pain are typical symptoms for various reasons, including physical effort, stress, and many medical disorders [44]. One study showed that the wild-type *TaqI* genotype was associated with reduced bone mineral density, which can lead to chronic fatigue and muscular pain, in North Indian women with osteoporosis [19]. Massidda et al. investigated the link between *VDR* genetic variants and muscle injury in 54 professional Italian football players; their study showed that only the *ApaI* genotype contributed to 18% of cases of severe muscle damage (*p* = 0.002) [45]. A study published in 2022 revealed that the homozygous genotypes of the *VDR ApaI* and wild-type *FokI* variants were linked to muscle pain and weakness in patients with fibromyalgia [46]. Furthermore, the metabolism of VD in airway epithelial cells increases airway hypersensitivity and influences the generation of inflammatory cytokines [47]. *VDR* polymorphisms are reportedly associated with stronger immune responses and decreased lung function, which may both lead to shortness of breath [20]. A previous study in Egypt showed that the heterozygous *TaqI* genotype was more frequently present in asthmatic children (*p* = 0.05), and there was no significant difference in *ApaI* genotype between patients and controls (*p* > 0.05) [48]. Furthermore, Papadopoulou et al. found substantial links between the homozygous *TaqI* genotype and persistent wheezing and active asthma severity [49].

This study had some limitations. First, the sample size was relatively small; thus, further clinical trials with larger sample sizes are needed. Second, although this study included the most common *VDR* genetic variants, rare variants excluded from the analysis may affect VD activity and influence COVID-19 severity. Lastly, it would be more precise to measure the serum inflammatory cytokines levels and COVID-19 inflammatory biomarkers to relate them to the severity of the symptoms. 

## 5. Conclusions

The *VDR TaqI* genotype may be associated with COVID-19 severity. Moreover, *VDR ApaI* and *TaqI* genotypes were significantly associated with persistent fatigue and muscle pain in a sample of Jordanian patients with COVID-19. Accordingly, *VDR ApaI* and *TaqI* variants may be associated with COVID-19 severity and long-COVID-19 symptoms in Jordanian individuals. However, further research involving larger populations across multiple ethnicities is needed to assess the potential impacts of diverse *VDR* genetic variants on the health consequences of long-COVID-19 symptoms.

## Figures and Tables

**Table 1:** Primer Sequences and Annealing Temperatures Used During PCR Amplification of the VDR Gene and the Expected DNA Fragment Sizes.

Primer	Primer Sequence (5′-3′)	Annealing Temperature (°C)	Size of DNA Fragment (bp)
*FokI F*	AGTTGGCCCTGGCACTGACTCTGCTCT	57	245
*FokI R*	ATGGAAACACCTTGCTTCTTCTCC CTC		
*ApaI-TaqI F*	CAGAGCATGGACAGGGAGCAA	55	745
*ApaI-TaqI R*	GCAACTCCTCATGGCTGAGGTCTC		

bp, base pair; F, forward primer; R, reverse primer.

**Table 2:** Demographics and Comorbidities of Patients With COVID-19.

Parameter	Value
Age (Mean ± SD)	37.39 ± 13.86 years
Sex Frequency (%)	Males 32 (32.0), Females 68 (68.0)
BMI frequency (%)	
Underweight	6 (6.0)
Normal weight	55 (55.0)
Over weight	36 (36.0)
Obese	3 (3.0)
Smoking habits frequency (%)	
Light smoker	19 (19.0)
Moderate smoker	9 (9.0)
Heavy smoker	2 (2.0)
Non-smoker	70 (70.0)
Comorbidities frequency (%)	
Type 2 diabetes	13 (13.0)
Hypertension	11 (11.0)
Chronic vascular disease	5 (5.0)
Lung disease	0
Chronic liver disease	0
Chronic kidney disease	0
Cancer	0
Pregnancy	3 (4.4) ^a^

^a^ Pregnancy (%) among female patients with COVID-19 (n = 68).

**Table 3:** Patient COVID-19 Status.

Frequency of Severity of COVID-19 Infection (%)	
Asymptomatic infection	50 (50.0)
Mild illness	48 (48.0)
Moderate illness	1 (1.0)
Severe illness	1 (1.0)
Critical illness	0
**Frequency of long-lasting Symptoms (%)**	
Fatigue and Muscle pain	27 (27.0)
Joint pain	22 (22.0)
Cough	8 (8.0)
Palpitation	2 (2.0)
Chest pain	3 (3.0)
Shortness of breath	7 (7.0)

**Table 4:** Frequencies of VDR *ApaI*, *FokI*, and *Taq1* Genotypes Among Patients with COVID-19.

*VDR* Genetic Variance Frequency (%)			
Genotype	*Taq1*	*FokI*	*ApaI*
Wild-type	36 (36.0%)	62 (62.0%)	54 (54.0%)
Heterozygous	45 (45.0%)	28 (28.0%)	32 (32.0%)
Homozygous	19 (19.0%)	10 (10.0%)	14 (14.0%)
Allele frequency (95% CI)			
Observed	0.41 (0.313–0.506)	0.24 (0.156–0.323)	0.30 (0.210–0.389)

**Table 5:** The *VDR* Haplotype Among Patients With COVID-19.

*VDR* Haplotype			
*ApaI*	*FokI*	*Taq1*	Frequency ^a^
C	C	T	0.282
A	C	C	0.235
A	C	T	0.228
C	T	T	0.111
A	T	T	0.078
A	T	C	0.043
C	C	C	0.014
C	T	C	0.007

**^a^** The Frequency of *VDR Apa1*, *FokI*, and Taq1 among patients infected with COVID-19 using the Haploview program.

**Table 6:** Associations of *VDR ApaI*, *FokI*, and *TaqI* Variants With COVID-19 Severity.

*VDR* Variant	Severity of COVID-19 Infection				
*ApaI* Genotype	Asymptomatic Infection	Mild Illness	Moderate Illness	Severe Illness	Critical Illness
Wild-type	21	15	0	0	0
Heterozygous	22	22	0	1	0
Homozygous	7	11	1	0	0
*p*-value	0.311	0.512	0.116	0.539	
*FokI* genotype	Asymptomatic infection	Mild illness	Moderate illness	Severe illness	Critical illness
Wild-type	31	29	1	1	0
Heterozygous	15	13	0	0	0
Homozygous	4	6	0	0	0
*p*-value	0.762	0.725	0.734	0.734	
*TaqI* genotype	Asymptomatic infection	Mild illness	Moderate illness	Severe illness	Critical illness
Wild-type	24	29	1	0	0
Heterozygous	17	15	0	0	0
Homozygous	9	4	0	1	0
*p*-value	0.38	0.242	0.65	0.045 *	
OR (95% CI)	-	-	-	19 (0.7–46)	-

* Statistical significance was defined as *p* < 0.05. OR represents odds ratio and CI denotes confidence interval.

**Table 7:** Associations of *ApaI*, *FokI*, and *TaqI* Genotypes With Long-COVID-19 Symptoms.

*VDR* Variant	Long-Lasting COVID-19 Symptoms					
*ApaI* Genotype	Fatigue and Muscle Pain	Joint Pain	Cough	Palpitation	Chest Pain	Shortness of Breath
Wild-type	3	5	0	0	1	3
Heterozygous	13	11	5	2	1	3
Homozygous	11	6	3	0	1	1
*p*-value	<0.001 *	0.279	0.071	0.287	0.805	0.908
OR (95% CI)	64 (11–350)	-	-	-	-	-
*FokI* genotype	Fatigue and Muscle pain	Joint pain	Cough	Palpitation	Chest pain	Shortness of breath
Wild-type	18	15	6	2	3	5
Heterozygous	5	6	1	0	0	2
Homozygous	4	1	1	0	0	0
*p*-value	0.337	0.601	0.595	0.535	0.388	0.65
*TaqI* genotype	Fatigue and Muscle pain	Joint pain	Cough	Palpitation	Chest pain	Shortness of breath
Wild-type	20	13	5	1	1	2
Heterozygous	1	7	2	0	1	1
Homozygous	6	2	1	1	1	4
*p*-value	0.036 *	0.733	0.877	0.28	0.585	0.003 *
OR (95% CI)	0.12 (0.04–0.41)	-	-	-	-	9 (0.9–86)

* Statistical significance was defined as *p* < 0.05. OR denotes odds ratio and CI represents confidence interval.

## Data Availability

Data are available from the corresponding author upon request.

## Supplementary Materials

The following supporting information can be downloaded at: https://www.mdpi.com/article/10.3390/jpm13121663/s1, Figure S1: Gel electrophoresis of RFLP products of *ApaI*, *FokI,* and *TaqI* polymorphisms. (a) Genotyping results for VDR *ApaI*; (b) Genotyping results for VDR *FokI*; (c) Genotyping results for VDR *TaqI*. Hetero, Heterozygous; Homo, Homozygous; W, Wild-type; DL, DNA loading ladder; Table S1: Distribution of Severity among Patients’ Characteristics other than VDR genotyping.
