# Supplementary material for: Exploring the Influence of VDR Genetic Variants TaqI, ApaI, and FokI on COVID-19 Severity and Long-COVID-19 Symptoms
Source: J Pers Med. 2023 Nov 28;13(12):1663. doi: 10.3390/jpm13121663 (PMC10744884; doi:10.3390/jpm13121663)
Supplement: Supplementary file 1 [file jpm-13-01663-s001.zip › jpm-2701568-supplementary.pdf]

Supplementary Table S1. Distribution of Severity among Patients' Characteristics other than VDR genotyping

Variable	Asymptomatic N (%)	Minor Illness N (%)	Moderate Illness N (%)	Sever Illness N (%)	*P* Value
**Gender**					0.269
**Male**	21 (41.2)	12 (25.0)	0 (0.0)	0 (0.0)	
**Female**	30 (58.8)	36 (75.0)	1 (100.0)	1 (100.0)	
**Age (Years Old)**					**0.000**
**Young Age (15 – 47)**	42 (82.4)	32 (66.7)	0 (0.0)	1 (0.0)	
**Middle Age (48 – 63)**	9 (17.6)	14 (29.2)	0 (0.0)	0 (0.0)	
**Old Age (64 and older)**	0 (0.0)	2 (4.2)	1 (100.0)	0 (0.0)	
**BMI**					**0.000**
**Normal Weight**	29 (56.9)	26 (54.2)	0 (0.0)	0 (0.0)	
**Underweight**	2 (3.9)	5 (10.4)	0 (0.0)	0 (0.0)	
**Overweight**	19 (37.3)	16 (33.3)	1 (100.0)	0 (0.0)	
**Obese**	1 (2.0)	1 (2.1)	0 (0.0)	1 (100.0)	
**Smoking Level**					0.995
**Non-Smoking**	34 (66.7)	34 (70.8)	1 (100.0)	1 (100.0)	
**Light Smoking**	12 (23.5)	8 (16.7)	0 (0.0)	0 (0.0)	
**Moderate Smoking**	4 (7.8)	5 (10.4)	0 (0.0)	0 (0.0)	
**Heavy Smoking**	1 (2.0)	1 (2.1)	0 (0.0)	0 (0.0)	
**Diabetes Mellitus**					0.086
**Yes**	6 (11.8)	7 (14.6)	1 (100.0)	0 (0.0)	
**No**	45 (88.2)	41 (85.4)	0 (0.0)	1 (100.0)	
**Hypertension?**					0.051
**Yes**	5 (9.8)	6 (12.5)	1 (100.0)	0 (0.0)	
**No**	46 (90.2)	42 (87.5)	0 (0.0)	1 (100.0)	

**Cardiovascular Disease?**					**0.001**
Yes	2 (3.9)	3 (6.3)	1 (100.0)	0 (0.0)	
No	49 (96.1)	45 (93.8)	0 (0.0)	1 (100.0)	
**Comorbidities?**					0.098
Yes	7 (13.7)	12 (25.0)	1 (100.0)	0 (0.0)	
No	44 (86.3)	36 (75.0)	0 (0.0)	1 (100.0)	

* P. values obtained from Chi-Square Test
* Bold *P* values are statistically significant (<0.05).

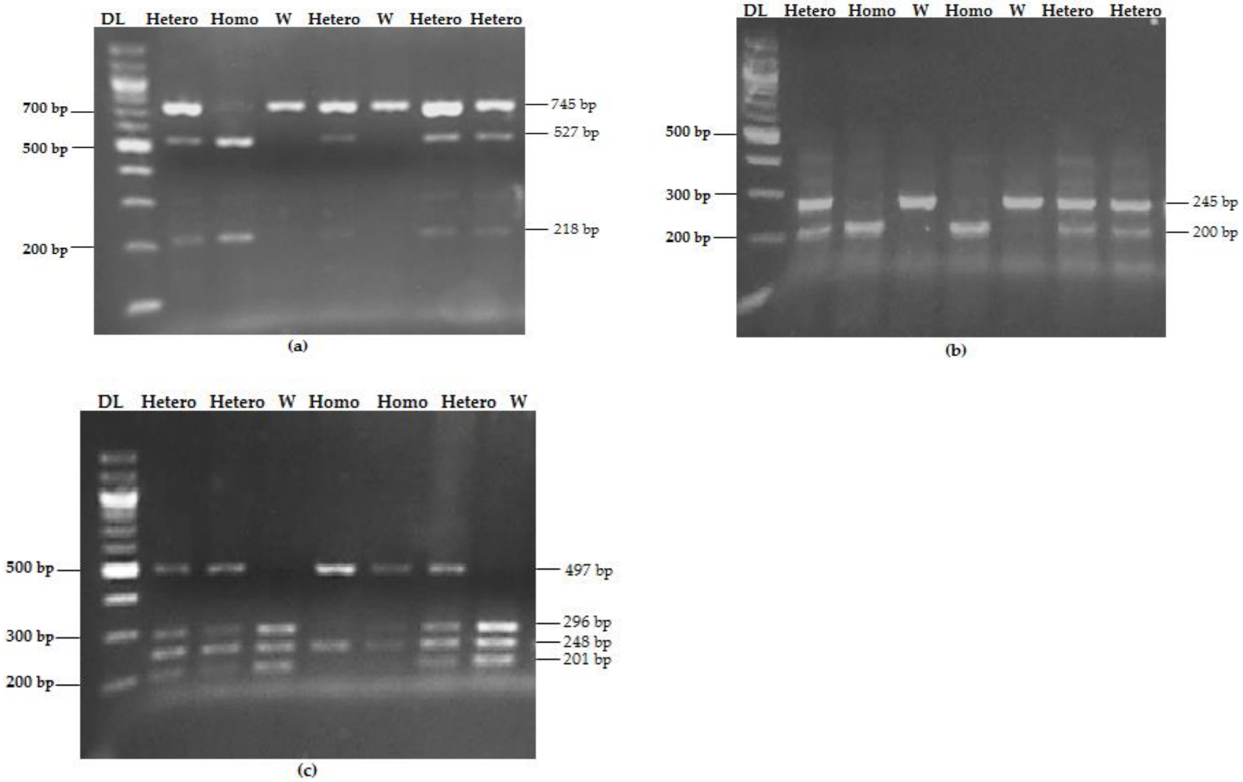

Supplementary Figure S1. Gel electrophoresis of RFLP products of *ApaI*, *FokI*, and *TaqI* polymorphisms. (a) Genotyping results for VDR *ApaI*; (b) Genotyping results for VDR *FokI*; (c) Genotyping results for VDR *TaqI*. Hetero, Heterozygous; Homo, Homozygous; W, Wild-type; DL, DNA loading ladder.
